# Mechanically and biologically skin-like elastomers for bio-integrated electronics

**DOI:** 10.1038/s41467-020-14446-2

**Published:** 2020-02-27

**Authors:** Shuo Chen, Lijie Sun, Xiaojun Zhou, Yifan Guo, Jianchun Song, Sihao Qian, Zenghe Liu, Qingbao Guan, Eric Meade Jeffries, Wenguang Liu, Yadong Wang, Chuanglong He, Zhengwei You

**Affiliations:** 1 State Key Laboratory for Modification of Chemical Fibers and Polymer Materials, Shanghai Belt and Road Joint Laboratory of Advanced Fiber and Low-dimension Materials (Donghua University), College of Materials Science and Engineering, Donghua University, Shanghai, 201620 PR China; 2 College of Chemistry, Chemical Engineering and Biotechnology, Donghua University, Shanghai, 201620 PR China; 3 School of Materials Science and Engineering, Tianjin Key Laboratory of Composite and Functional Materials, Tianjin University, Tianjin, 300350 PR China; 4 Meinig School of Biomedical Engineering, Cornell University, Ithaca, NY 14853 USA

**Keywords:** Electrical and electronic engineering, Bioinspired materials, Polymers

## Abstract

The bio-integrated electronics industry is booming and becoming more integrated with biological tissues. To successfully integrate with the soft tissues of the body (eg. skin), the material must possess many of the same properties including compliance, toughness, elasticity, and tear resistance. In this work, we prepare mechanically and biologically skin-like materials (PSeD-U elastomers) by designing a unique physical and covalent hybrid crosslinking structure. The introduction of an optimal amount of hydrogen bonds significantly strengthens the resultant elastomers with 11 times the toughness and 3 times the strength of covalent crosslinked PSeD elastomers, while maintaining a low modulus. Besides, the PSeD-U elastomers show nonlinear mechanical behavior similar to skins. Furthermore, PSeD-U elastomers demonstrate the cytocompatibility and biodegradability to achieve better integration with tissues. Finally, piezocapacitive pressure sensors are fabricated with high pressure sensitivity and rapid response to demonstrate the potential use of PSeD-U elastomers in bio-integrated electronics.

## Abstract (web summary)

Designing biodegradable and biocompatible polymers to mimic both mechanical and biological properties of skins for emerging electronic devices remains a challenge. Here, the authors propose PSeD-U skin-like elastomers with both mechanical and biological properties for bio-integrated electronics.

## Introduction

Electronics are rapidly merging with biology through the increased prevalence of bio-integrated electronics in wearable electronics^1–4^, robotics^5^, electronic skin^6–8^, human–machine interfacing^9–11^, and implantable electronics^12,13^. Despite rapid progress in the capability and miniaturization of integrated circuits technology, the mechanical, and biological design in bio-integrated electronics remains limited^6,14^. Electronics using rigid substrates and brittle components lack of biocompatibility and biodegradability, resulting in mechanical mismatch and complications in the contact areas between the devices and the surrounding tissues^15^. Skin is the largest organ of the human body with soft and tough mechanical performance^16^. The major components of skin are collagen and elastin fibers^17^. Based on the cooperation of these two mechanically distinctive components, the mechanical behavior of the skin exhibits a nonlinear stress–strain relationship with low modulus, self-stiffness, high toughness, and tear-resistance^16^. Similarly, these mechanical properties are also desirable for conformability and self-protection of bio-integrated electronics especially electronic skin and wearable electronic devices^6^. However, it remains a challenge to develop elastic materials with a suitable combination of nonlinear viscoelasticity, toughness, softness, and stretchability to mimic skin’s mechanical properties^18–20^. Some elastic materials, such as poly(dimethylsiloxane) (PDMS), polyurethane, polystyrene-block-poly(ethylene-ran-butylene)-block-polystyrene, have been used to fabricate stretchable electronic devices^15,21–24^. However, these elastomers cannot mimic the mechanical properties of human skins and generally have high elastic modulus and low toughness^12,15^. Fiber reinforced composites have been developed to mimic the mechanical properties of skin. The nanofibers increased the toughness of resultant composites, but also compromised the stretchability (below 150%)^25,26^. The lack of strong interaction between nanofibers and matrix may lead to stability issues when the elastic materials were subjected to cyclic deformation. Thus, it remains a challenge to develop elastic materials with a suitable combination of nonlinear viscoelasticity, stretchability, toughness, and softness to mimic skin’s mechanical properties. Very recently Sheiko et al. reported a synthetic elastomer mimicking the mechanical properties of organ tissues with brush and comb-like polymer networks^18^. However, the structure of the elastomer is complicated and challenging to fabricate. Furthermore, most of the previous works only focused on the mechanical properties, but overlooked the biological properties of the human skins, which are highly desired for improved interfacing with wearable and transient electronics^12,27^. Therefore, it is urgent to develop a new class of electronic elastomers that mimics both the mechanical and biological properties of skins for emerging electronic devices^8^.

**Fig. 1:**
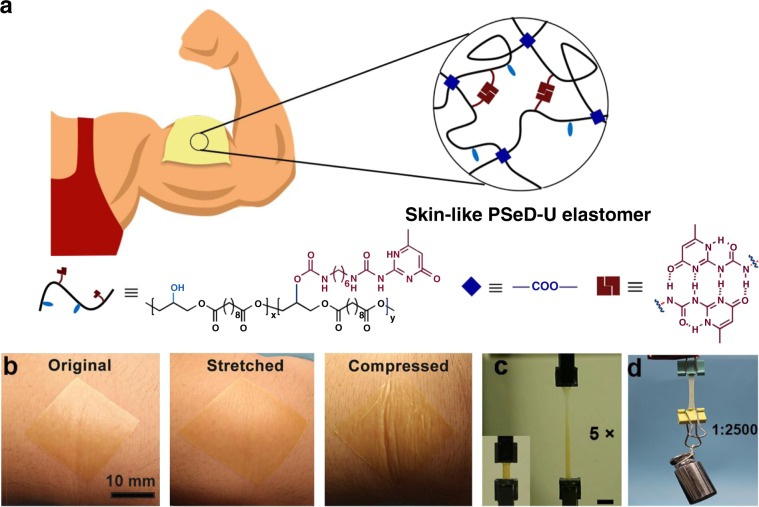
Schematic and performance of skin-like PSeD-U elastomers. **a** The schematic illustration of the skin-like PSeD-U elastomers with physical and covalent hybrid crosslinked structures. **b** Photographs of spin-coated ultrathin PSeD-U20 film (70 µm thick) attached onto human skin: original, stretched, and compressed, illustrated its high conformability. Scale bar = 10 mm. **c** Photographs of original and five times stretched PSeD-U20-12h elastomer specimen showed the high stretchability of PSeD-U elastomers. Scale bar = 10 mm. **d** PSeD-U20-12h elastomer (40 mg) could hold a weight of 100 g (2500× its own weight) revealing its high strength and toughness.

To address aforementioned challenges, we aimed to develop new mechanically and biologically skin-like elastomers. Recently, introducing physical interactions into the covalent networks to form dual-crosslinked networks, so called sacrificial bonds, has been proven an effectively strategy to enhance the materials’ toughness^28–32^. Ideally, the introduced physical interactions would have no discernable impact on the covalently crosslinked polymer chains’ mobility at small deformation, while permitting the physical interactions to achieve strain-induced dissociation for mechanical energy dissipation at large deformation^33,34^. The resultant elastomers would keep the modulus of the covalently crosslinked networks and obtain superior toughness by the energy dissipation effect of sacrificial physical interactions. To mimic the biological properties of skins as well, biodegradable and biocompatible polymers with well-defined functional groups enabling controllable introduction of physical and covalent crosslinking are desired. We previously developed the acid-induced epoxide ring-opening polymerization to readily produce functional biodegradable and biocompatible polyesters with free hydroxyl groups, which can be used as synthetic platform for this work^35^. Accordingly, we design the poly(sebacoyl diglyceride) (PSeD)-graft-2-ureido-4[1H]-pyrimidi-none unit (UPy) marked as PSeD-U, by incorporating the physical hydrogen bonds and the covalent crosslinks to form unique hybrid crosslinked networks (Fig. 1a). The covalent crosslink density can be readily controlled by the degree of esterification of hydroxyl group. The physical crosslinking was modulated by incorporating biocompatible UPy units with self-complementary quadruple hydrogen bonds via free hydroxyl groups^36^. In this study, the PSeD-U elastomers were successfully synthesized and demonstrated their skin-like mechanical properties as well as biocompatibility and biodegradability. Furthermore, we design and fabricate highly sensitive piezocapacitive pressure sensors based on the microstructured PSeD-U dielectric component, demonstrating its potential in bio-integrated electronics.

## Results

### Synthesis and characterization of PSeD-U elastomers

As designed we prepared a series of PSeD-U elastomers with different concentrations of UPy groups (named as PSeD-Ux, where x means the certain equiv of UPy groups based on the theoretical amounts of hydroxyl groups) (Supplementary Figs. 1 and 2). The increased molecular weights of PSeD-U polymers compared with PSeD confirmed the efficiency of these reactions (Supplementary Table 1). The structures of resultant polymers were confirmed by NMR spectroscopy (Supplementary Fig. 3). The actual UPy contents in PSeD-U polymers were close to the theoretical contents evidenced by comparing the relative proton integrations of the pyrimidyl groups in UPy at *δ* 5.71 ppm and the methylene groups in sebacate at *δ* 2.27 ppm (Supplementary Table 1). The inductively coupled plasma (ICP) tests were performed to further confirm the actual UPy concentration in the PSeD-U elastomers (Supplementary Table 2). The reaction efficiency and polymer structures were further confirmed by attenuated total reflectance Fourier transformed infrared (ATR-FTIR) (Supplementary Fig. 4). The PSeD-U polymers were thermally cured for predetermined times to obtain various hybrid crosslinked elastomers (e.g., PSeD-U20-12h means that 12-h cured PSeD-U20). The wide angle X-ray diffraction (WXRD) patterns of these PSeD-U elastomers only displayed a broad amorphous diffraction peak at a 2*θ* of 21°, suggesting that they are all amorphous at room temperature (Supplementary Fig. 5). To further clarify the hybrid crosslinked structures of our system, we performed the rheological tests of both PSeD and PSeD-U elastomers by measuring the storage and loss moduli at different frequencies under constant low strain (0.5%) (Supplementary Fig. 6). The storage modulus (G′) of PSeD-12h and PSeD-U20-12h elastomers were higher than their loss modulus (G″) across the measured frequency range, demonstrating their covalently crosslinked molecular structures^37,38^. The G′ of PSeD-12h elastomers remained relatively constant across the frequency range due to the stable covalent crosslinked network. Compared with PSeD-12h elastomers, the PSeD-U20-12h elastomers showed a gentle frequency-dependent trend partly caused by the dynamic hydrogen bonds. However, the storage modulus of PSeD-U20-12h increased slowly with the frequency and was slightly higher than that of PSeD-12h elastomers, which indicated the influence of hydrogen bonds to the storage modulus was limited. Meanwhile, the G″ exhibited a frequency-dependent trend for PSeD-U elastomers, indicating that the sacrificial hydrogen bonds have a more marked effect on the G″^34^. With the frequency increased to approach the hydrogen bonds kinetics, the dissociation of hydrogen bonds and chain relaxation produced energy dissipation and led to higher G″ of PSeD-U20-12h than that of PSeD-12h elastomer. Based on the unique hybrid crosslinked structures, the PSeD-U elastomers showed soft, tough, and strong mechanical performance. When attached onto human skins, the ultrathin PSeD-U elastomers film (70 µm) could sustain the stretch and compression of natural human motions and even show a conformal attachment to curved skin and wrinkled surfaces (Fig. 1b). Such an ultrathin film structure and soft nature allow the PSeD-U elastomer to be stretched and compressed with the skin freely and reversibly, leading to the stable adhesion and conformability. The PSeD-U elastomers showed large stretchability (Fig. 1c) and strong, tough mechanical performance similar to human skin (Fig. 1d). The superior mechanical performance permitted the skin-like elastomers to be easily fabricated into ultrathin films, which were able to be handled without tearing^25^.

### The skin-like mechanical properties of PSeD-U elastomers

**Fig. 2:**
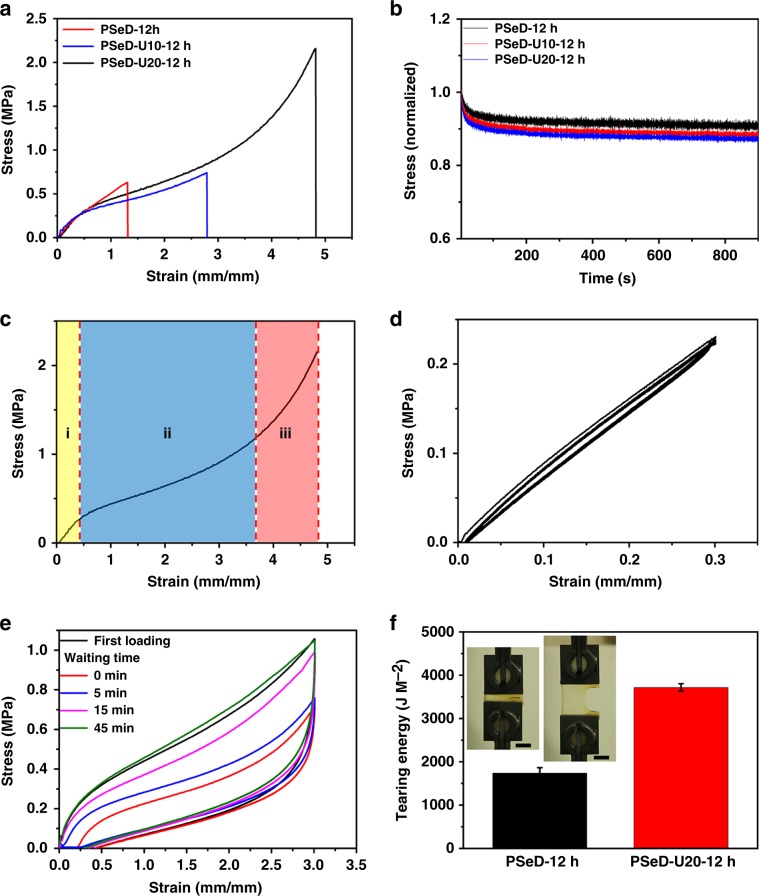
Skin-like mechanical properties of PSeD-U elastomers. **a** Typical tensile stress–strain curves of PSeD-U-12h elastomers with different densities of hydrogen bond while practically identical covalent crosslinking densities. **b** The stress relaxation curves of samples stretched at 5% strain for 900 s. **c** Dependence of stress on the strain, the stress–strain curve can be divided into three regions depending on the ranges of strain. **d** Cyclic tensile tests with a strain of 30%. **e** The tensile stress–strain curves of loading-unloading cycles with 300% strain with different waiting time between two consecutive loadings. **f** The comparison of tear energy of PSeD-12h and PSeD-U20-12h. The insert photograph is a notched PSeD-U20-12h specimen with a strain of 300%, showing its high tear resistance. Scale bar = 10 mm.

The mechanical properties of PSeD-U20 elastomers with different covalent crosslink densities were examined by adjusting the curing times (Supplementary Fig. 7). The elastomers with different covalent crosslinks densities were quantitatively confirmed by their corresponding swelling ratios in the N,N-Dimethylformamide (DMF) (Supplementary Table 3). In view of the mechanically biomimetic properties, the PSeD-U20-12h elastomers had the modulus (0.64 ± 0.10 MPa) closest to the skin (0.42-0.75 MPa)^39^, and a relatively high toughness. Thus, the 12-h curing time was chosen for further test. The effect of UPy groups on the mechanical properties of elastomers were then evaluated (Fig. 2a and Supplementary Fig. 8). Compared with PSeD-12h elastomers, the tensile strength (0.73 ± 0.10 MPa) and maximum elongation (297 ± 16%) of PSeD-U10-12h increased by 1.14 and 2.18 times, while keeping a similar Young’s modulus. With the increase of UPy groups (PSeD-U20-12h), the tensile strength and maximum elongation further increased to 2.42 ± 0.54 MPa and 509 ± 24% while displaying an almost constant Young’s modulus of 0.64 ± 0.10 MPa (Fig. 2a). The toughness (the area under strain–stress curves) of PSeD-U20-12h was improved by more than 11 times compared with PSeD-12h elastomers and higher than the other reported PGS-based elastomers (Supplementary Fig. 9), demonstrating the effective energy dissipation capability of hydrogen bonds in PSeD-U elastomers. It is worth noting that sacrificial hydrogen bonds do not contribute to the initial stiffness, as the Young’s moduli of these elastomers were all near 0.60 MPa. This phenomenon is in accordance with the previous studies of “mechanically invisible” polymeric materials^34^, where the physical sacrificial bonds in materials that did not contribute to the initial stiffness but sufficiently dissipated mechanical energy with an increased elongation and stress. Presumably, in these elastomers, the Young’s modulus was mainly determined by the covalent crosslinking density and polymer chains entanglements rather than the low concentration of hydrogen bonds, which made the hydrogen bonds “mechanically invisible” at initial deformation. According to this assumption, when the hydrogen bond concentration increased to a certain percentage, hydrogen bonds would contribute to initial modulus as well. Accordingly, the PSeD-U30-12h elastomer showed a significant higher Young’s modulus (1.49 ± 0.24 MPa), likely due to the stronger hydrogen bonds induced by the UPy units and carbamate linkages in increased UPy-HDI groups (Supplementary Fig. 10). These results confirmed our hypothesis and indicated the relatively low concentration of hydrogen bonds under a certain critical value is the key point to keep the low modulus. The stress-relaxation experiments at constant strains were performed to further confirm the role of hydrogen bonds in Young’s modulus. At small strain (5%), the drop of the stress of PSeD-U10-12h and PSeD-U20-12h elastomers were very limited, similar to that of PSeD-12h elastomer (Fig. 2b). These results were evidence that the hydrogen bonds of these PSeD-U elastomers had limited effect on the initial modulus. However, at large strain (300%), the release of applied force of PSeD-U20-12h elastomers was much higher than the one at small strain, indicating the energy dissipation resulted from the strain-dependence dissociation of hydrogen bonds (Supplementary Fig. 11). Furthermore, the elastomeric networks were estimated using classical Neo-Hookean model with the assumption that the polymer chains of the elastomers were Gaussian chains and that the microscopic deformation of the polymer chains of the elastomers was proportional to the macroscopic deformation of the elastomer (Supplementary Fig. 12). The similar modulus also indicated the introduction of low concentration of hydrogen bonds would have little effect on the average molecular weight between the crosslinking points^20^. To study the probable mechanism of mechanical behavior of PSeD-U elastomers, the stress–strain curve was divided into three regions depending on the different ranges of deformation (Fig. 2c and Supplementary Fig. 13)^19^. At low strain (region i), and the sacrificial hydrogen bonds were “mechanically invisible,” the PSeD-U20-12h elastomers showed a modulus similar to PSeD-12h elastomers. With the increase of strain, the hydrogen bonds dissociated to dissipate mechanical energy. As a result, the corresponding slope of strain–stress curves in region ii become smaller than region i. At large strain (region iii), the hydrogen bond dissociation likely saturated. The major contribution of the resistance of the applied strain was the covalent crosslinked networks. Accordingly, the modulus of region iii increased rapidly compared with region ii. This unique mechanical profile of nonlinear stress–strain behaviors with self-stiffening properties during stretching of PSeD-U20-12h elastomer was similar to that of human skin^16^.

The cyclic tensile tests at different strains were performed to further demonstrate the aforementioned mechanism. The loading and unloading curves of PSeD-U20-12h elastomers at low strain (30%) were superimposable and showed negligible hysteresis (Fig. 2d). It is worth noting that the fast deformation and recovery at low strain (10–50%) is a desirable and important property for sensory and actuating applications, such as artificial muscles^40^, skin-like sensors^41^, and tissue–electronic interfaces^42^. With the increase of strain, the cyclic tensile curves were no longer superimposable and hysteresis started to develop. This indicated that part of the applied mechanical energy during stretching process was dissipated (Supplementary Fig. 14). The cyclic tensile curve at 300% strain of PSeD-U20-12h elastomers showed an obvious hysteresis loop, but completely recovered to the original curve after a relatively short waiting time (∼45 min) (Fig. 2e). The recovery process showed an interesting two-stage process due to the competition between the contractile force of the elastic covalent network pulling against temporary hydrogen bonds (Supplementary Fig. 15). While the resistance force of hydrogen bonds was likely constant. The contractile force from covalent network was proportional to the deformation. Larger deformation led to stronger elastic contraction, which efficiently dissociated the reformed hydrogen bonds and resulted in faster recovery. Smaller deformation had relatively weaker elastic contraction, which was significantly hindered by reformed hydrogen bonds, resulting in slower recovery. The cyclic tensile tests were performed to evaluate the robustness of PSeD-U20-12h elastomers (Supplementary Fig. 16). The repeatable stress–strain curve and toughness demonstrated the robust recovery ability of PSeD-U elastomers.

Tear resistance is also one of the important features of skin, especially providing the protection from external attack^43^. Similarly, the tear resistance of elastomers used in electronics is also important for the protection of integrated circuits for wearable electronics. However, the previous reported skin-inspired elastomers rarely investigated it. Rivlin–Thomas pure shear tests were performed to evaluate the tear energy of the PSeD-U20-12h elastomers (Fig. 2f and Supplementary Fig. 17). The PSeD-U20-12h elastomer with a cut notch could be successfully stretched to 3.5 times and showed a tearing energy of *T* = 3670 ± 86.6 J/m^2^, improved two times compared with PSeD-12h elastomers (1738 ± 125.0 J/m^2^), indicating the efficient energy dissipation of hydrogen bonds. Furthermore, the tear energy of PSeD-U20-12h elastomers was comparable with skin (up to 3600 J/m^2^)^44^ and almost four times higher than widely used electric elastomer PDMS (960 J/m^2^)^45^.

### The biological properties of PSeD-U elastomers

**Fig. 3:**
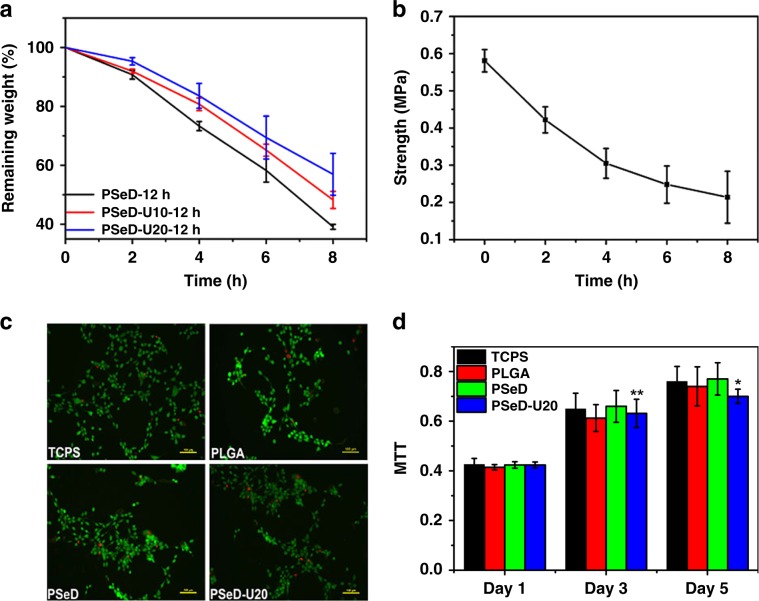
The degradation behaviors and cytocompatibility of PSeD-U elastomers. **a** In vitro enzymatic degradation of PSeD and PSeD-U elastomers in lipase DPBS solutions (0.1 M) at 37 °C. **b** Tensile strength of PSeD-U20-12h at a strain of 200% after degradation for 0, 2, 4, 6, 8 h, showing a gradual decrease of mechanical strength. **c** Images of NIH 3T3 fibroblasts in live/dead assays of TCPS, PLGA, PSeD, and PSeD-U20. A large amount of live cells (green) and few dead cells (red) at all surface, indicating the good biocompatibility of PSeD-U (magnification = ×100; scale bar = 100 μm). **d** The MTT assay of NIH 3T3 fibroblasts cells cultured on TCPS, PLGA, PSeD, and PSeD-U20 elastomers. There was no significant difference between different materials at the same time point. Statistical significance compared with previous time point was marked as *p < 0.05 or **p < 0.01.

Biological properties, such as biodegradability and biocompatibility, are important characteristics for human skins and highly desired for skin-like elastomers and wearable electronics^8,46^. However, few studies reported biodegradable and biocompatible skin-like elastomers. The in vitro enzymatic degradation of PSeD-U elastomers was evaluated in 0.1 M dulbecco’s phosphate-buffered saline solutions and 2000 U/mL lipase at 37 °C (Fig. 3a). During the tests, all the samples maintained their original rectangle shapes and exhibited a gradual decrease in dimension. Compared with PSeD-12h, PSeD-U10-12h, and PSeD-U20-12h degraded much slower with a remaining weight of 48.2% ± 2.9% and 56.9% ± 7.1% after 8 h, respectively, which demonstrated that the strong hydrogen bonds between UPy units slowed the degradation of PSeD-U elastomers. Similar phenomenon was observed in the degradation test without lipase (Supplementary Fig. 18). More importantly, the tensile strength of PSeD-U elastomers showed a linearly gradual decrease with time, indicating their good ability to retain mechanical integrity during degradation (Fig. 3b and Supplementary Fig. 19).

The cytocompatibility of PSeD-U polymers was evaluated by seeding NIH 3T3 fibroblasts on PSeD-U20. The tissue culture polystyrenes (TCPS), unmodified PSeD, and poly(lactic-co-glycolic acid) (PLGA) were used as controls. After 48 h, live/dead assays clearly showed a large number of live cells (green) and few dead cells (red) at all surface (Fig. 3c). 3-(4,5-Dimethylthiazol-2-yl)-2,5-diphenyltetrazolium bromide (MTT) assay was conducted to evaluate the fibroblasts proliferation on PSeD-U20 (Fig. 3d). The number of metabolically active fibroblasts on PSeD-U20 significantly increased with the time of culture and was not statistically different from those on TCPS, PLGA, and PSeD at the same time points. All these data suggested that PSeD-U20 had a good in vitro biocompatibility, which is a key point for biomedical applications.

Subcutaneous implantation in mice was performed to evaluate the in vivo biocompatibility of PSeD-U20-12h elastomers using PLGA as the control (Supplementary Fig. 20). After the implantation, all mice were in good condition and no infections or abscesses were observed. The body weights of mice kept relatively constant during the period of experiments, which indicated the implantation had no adverse effect on the growth of mice (Supplementary Fig. 20a). The hematological test was performed to further detect inflammatory reaction (Supplementary Fig. 20b). The values of white blood cell (WBC) concentration in PSeD-U20-12h elastomers and PLGA groups were all similar during the experimental period. After 7 days of implantation, both showed a slight increase compared with normal mice without implantation. That was mainly owing to the surgical wound inflammation^47^. In the following retrieving time points (14 and 28 days), the value of WBC decreased to normal level. To further evaluate the in vivo biocompatibility of the PSeD-U20-12h elastomers, the short-term and long-term host responses to implants were investigated via H&E staining (Supplementary Fig. 20c). It was found that the elastomers were surrounded by loose connective tissue, indicating the mild inflammation. These results demonstrated that PSeD-U20-12h elastomers exhibited comparable in vivo biocompatibility with the commonly used biomaterial PLGA. The implants were retrieved at different time points after implantation (Supplementary Fig. 21). Compared with PLGA, the sizes of PSeD-U20-12h elastomers showed obvious change with the increase of implantation time, demonstrating the good biodegradability of PSeD-U20-12h elastomers.

### The application of PSeD-U elastomers for bio-integrated sensors

**Fig. 4:**
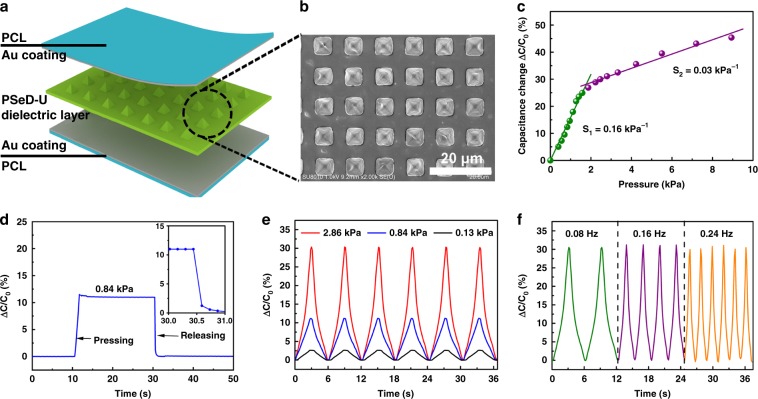
Schematic and characterization of PSeD-U elastomers based piezocapacitive pressure sensor. **a** Schematic of the structure of the piezocapacitive pressure sensor. **b** SEM images of the microstructured PSeD-U20-12h elastomer dielectric film. **c** Pressure–response curve of the piezocapacitive pressure sensor. **d** Response times of the pressure sensor with the pressure of 0.84 kPa. The inset shows the response time of the pressure sensor to pressure release. **e** Dynamic responses of the pressure sensor at various pressures with a frequency of 0.08 Hz. **f** Dynamic responses of the pressure sensor at various frequencies with a pressure of 2.86 kPa.

In recent years, soft, and flexible skin-like electronic devices have attracted more and more attentions and become a novel platform to integrate with soft tissues for human–machine interaction, health monitoring, and medical therapies^3,6,8,48^. Several skin-like sensors (e-skins, e-tattoos, epidermal device) based on different mechanisms, such as resistive, capacitive, piezoelectric, and triboelectric principles, have been developed and applied to the human mechanical sensing^15^. The materials with an elastic modulus ranging from 10 kPa to a few hundred kPa and robust elasticity (deform up to 15%) were developed to meet the mechanical requirement of skin electronics. Based on the skin-like electronic materials, the skin electronics were able to conform the topography of the skin and accommodate strains during natural body motion. To demonstrate the application of PSeD-U elastomers for bio-integrated electronics, we fabricated the piezocapacitive pressure sensor based on a microstructured PSeD-U20-12h elastomer dielectric film. Schematic illustration described the design principle of the piezocapacitive pressure sensor, which consists of a microstructured PSeD-U dielectric layer between the top and bottom electrodes (Fig. 4a). Figure 4b shows the SEM image of the microstructured surface of the PSeD-U dielectric layer with a thickness of around 100 µm, which were prepared by spin-coating PSeD-U20-12h elastomer onto a pyramidal recesses etched silicon wafer mold. The pyramids on the surface of PSeD-U20-12h elastomer had a height of 3.4 µm, a pyramid base width of 5 µm, and a spacing between pyramids of 5 µm. These pyramidal microstructures provided voids that allow the PSeD-U20-12h dielectric layer to elastically and reversibly deform in response to applied pressure. Gold was selected for the electrodes due to its biocompatibility^49^. A pressure sensor with a size of 2 × 2 cm^2^ was used to investigate the pressure sensitivity. The sensitivity (*S*) was defined as the slope of the curve in Fig. 4c (*S* = *δ*(Δ*C/C*_0_)*/δ*_*P*_), where *C* and *C*_0_ were the capacitances with and without applied pressure, and *p* was the applied pressure. The piezocapacitive sensor using the microstructured PSeD-U dielectric layer showed two different stages with different compressive behaviors upon an increase in the pressure input. The pressure sensitivity is 0.16 kPa^−1^ in a low-pressure regime (*p* < 2 kPa) and 0.03 kPa^−1^ at higher pressures (2 < *p* < 10 kPa). The low modulus of the PSeD-U20-12h elastomers and the reversible compressibility of the microscaled pyramids structure on the surface of dielectric layer were considered the key features for the sensitive pressure-sensing capability. It should be noted that the pressure sensitivity could be further improved by optimizing the parameters of microstructures. These pressure sensors showed rapid response to the applied pressure, especially in the pressure releasing stage. The response time for the return to the original baseline was in the millisecond range due to the excellent elasticity of the microstructured PSeD-U dielectric layer (Fig. 4d). The pressure sensors exhibited good stability and showed minimal hysteresis under cyclic compression test (Fig. 4e, f). The pressure sensor produced reversible pressure response curves with stable, noise-free, and successive output signals through cyclic compression testing at various pressures or frequencies. This excellent pressure-sensing performance of the PSeD-U elastomer pressure sensor would be very useful for human tactile pressure sensing. A pressure sensor was attached to the human throat and the capacitances change during human speak activities was recorded (Supplementary Fig. 22). When the person spoke the phrase of “DHU”, the relative capacitance change curves exhibited similar and repeated characteristic peaks, which demonstrated the promising applications of pressure sensors in human/machine interaction. Furthermore, to demonstrate the applications for stretchable electronics, skin-like strain sensors were prepared by spraying silver nanowires on PSeD-U elastomer films (Supplementary Fig. 23). The uniformly dispersed silver nanowires formed a conductive network, which showed a strain sensitive conductivity. Due to the skin-like softness and good stretchability, the strain sensor was capable of not only stretching to accommodate the strains of human motion but also deforming to conform the topography of the skin. As a result, the motion of wrist including bending and stretching was well detected by the change of the resistance of the sensor being attached to the back of a wrist.

## Discussion

To the best of our knowledge, the PSeD-U elastomers are the first example to mimic both mechanical and biological properties of skins in terms of nonlinear mechanical behavior, suitable softness, high strength, toughness and tear-resistance, and good biocompatibility and biodegradability. The skin-like mechanical properties were readily achieved by a combination of hydrogen bonds and a covalently crosslinked network to mimic the roles of collagen and elastin fibers in natural skins. The ratio between hydrogen bond and covalent crosslinks was found to play a critical role on the biomimetic mechanical properties. Adjusting this ratio alters the response to different strains during the deformation. This observation provided a new insight into the widely used dual crosslinked system. This work paves the way for a new, simple and powerful material design to mimic the sophisticated mechanical properties of natural soft tissues. In addition, the synthetic route based on versatile acid-induced epoxide ring-opening polymerization and consequent functionalization can be readily applied to diverse molecular structures and interactions. We expect this strategy will inspire a series of biomimetic materials for a wide range of applications, such as wearable electronics, biomimetic sensors, medical implants, tissue engineering scaffolds, human–machine interfacing, and soft robotics.

## Methods

### Synthesis of UPy-HDI

2-Amino-4-hydroxy-6-methylpyrimidine (17.024 mmol, 2.128 g) was mixed with 6 equiv of hexamethylene diisocyanate (102.14 mmol, 17.170 g). The mixture was put in a round-bottom flask in a glovebox filled with nitrogen. The flask was sealed and transferred out from the glovebox. The mixture was stirred, and heated at 100 °C under a nitrogen atmosphere for 16 h. The reaction mixture was washed by hexane three times to remove the unreacted hexamethylene diisocyanate. The product was dried at 50 °C under vacuum overnight to obtain white powder (4.690 g; yield, 94%).

### Synthesis of PSeD-U

Predetermined amount of UPy-HDI (0.1, 0.2, and 0.3 equiv of theoretical amount of hydroxyl groups in PSeD) and PSeD were mixed in anhydrous DMF in a Schlenk flask in a glovebox filled with nitrogen. The flask was sealed, transferred out of the glovebox, and connected with a Schlenk line. The mixture was heated to 100 °C and stirred under a nitrogen atmosphere for 36 h. The mixture was rotary evaporated under vacuum to remove the DMF and washed by ethyl ether three times to get a yellow solid.

### Fabrication of cross-linked PSeD-U elastomers

The hexafluoroisopropanol solution of PSeD-U at a concentration of 50% (w/v) were cast into a Teflon mold and dried in an oven at 40 °C for 3 days to slowly evaporate the solvent. Then the Teflon mold was transferred into a vacuum oven and thermally cured (130 °C) for predetermined time (6, 12, and 24 h) to obtain elastomers.

### General characterization

^1^H NMR spectra were recorded by Bruker Avance 600 NMR. ATR-FTIR spectra of the PSeD and PSeD-U polymers were recorded by a Thermol Nicolet 8700 spectrometer. The molecular weights of the polymers were determined by gel permeation chromatography (GPC) on a Viscotek/Malvern GPC system consisting of a GPCMax with a Dual 270 array (differential refractive index and right-angle light scattering, low-angle static light scattering, and four-capillary differential viscometer detectors) using dimethylacetamide as the eluent at a flow rate of 0.7 mL/min at 40 °C. WXRD measurements were carried out at room temperature on flat polymer films (length × width × thickness = 10 mm × 10 mm × 0.3 mm) using a Rigaku D/max-2550 PC instrument. The scanning range of the Bragg 2q angle varied from 3 to 60° at a scanning rate of 20°/min. Differential scanning calorimetry was performed on a TA Q 200 at a heating rate of 10 °C/min under a nitrogen atmosphere. The ICP tests were performed on a Leeman Prodigy ICP emission spectrometer.

## Supplementary information

Supplementary Information

## Data Availability

Data supporting the findings of this study are available within the paper and its Supplementary Information files. All other relevant data are available from authors upon reasonable request.
