# Supplementary material for: Mechanically and biologically skin-like elastomers for bio-integrated electronics
Source: Nat Commun. 2020 Feb 27;11:1107. doi: 10.1038/s41467-020-14446-2 (PMC7046662; doi:10.1038/s41467-020-14446-2)
Supplement: Supplementary file 1 — Supplementary Information [file 41467_2020_14446_MOESM1_ESM.pdf]

Supporting Information

for

Mechanically and biologically skin-like elastomers for bio-integrated electronics

Shuo Chen[1], Lijie Sun[1], Xiaojun Zhou[2], Yifan Guo[1], Jianchun Song[1], Sihao Qian[1], Zenghe Liu[2], Qingbao Guan[1], Eric Meade Jeffries[3], Wenguang Liu[4], Yadong Wang[5], Chuanglong He[2], Zhengwei You[1*]

[1]State Key Laboratory for Modification of Chemical Fibers and Polymer Materials, Shanghai Belt and Road Joint Laboratory of Advanced Fiber and Low-dimension Materials (Donghua University), College of Materials Science and Engineering, Donghua University, Shanghai 201620, P. R. China.

[2]College of Chemistry, Chemical Engineering and Biotechnology, Donghua University, Shanghai 201620, P. R. China.

[3]Unaffiliated: emjeffries@gmail.com

[4]School of Materials Science and Engineering, Tianjin Key Laboratory of Composite and Functional Materials, Tianjin University, Tianjin 300350 , P. R. China.

[5]Meinig School of Biomedical Engineering, Cornell University, Ithaca, NY, 14853, USA.

*Corresponding author. Email: zyou@dhu.edu.cn (Z.Y.)

Supplementary Methods

Rheological test

The rheological measurements were performed on a TA ARES-RFS. Frequency sweeps were performed at a constant strain of 0.5% by varying the frequency from 0.1 rad/s to 100 rad/s at 30 ^{o}C , respectively.

Swelling test

The swelling tests were used to estimate the covalent crosslinking density of the elastomer samples using the established method. The samples were swollen in DMF for 24 h, then swabbed using filter paper and weighted to determine m_e. The samples were then dried in a vacuum oven at 60 ^{o}C for 48 h and weight again to determine m_d. Swelling was quantitatively described by swelling percentage, defined as $(m_e - m_d)/m_d$.

Mechanical test

Dog bone-shaped specimens of PSeD and PSeD-U elastomers were made by melt molding and solvent cast using a HFIP solution of PSeD and PSeD-U polymers, respectively, in a Teflon mold (D412-06a die A design scaled by 1/4, length × width × thickness = 14.75 × 3 × 0.3 mm). Then, the Teflon mold was thermally cured (130 ^{o}C) for predetermined time to obtain bioelastomer samples. Tensile tests were performed on an MTS machine equipped with a 100 N loading cell at a deflection rate of 50 mm/min at room temperature. At least three specimens were tested and averaged for each material.

Stress relaxation

Stress relaxation tests were performed on MTS machine equipped with a 100 N loading cell. The samples were stretched to a small strain (5%) and a large strain (300%), then held for 900 s. The decreased stress was measured during the relaxation process.

Cyclic tensile test

The cyclic tensile tests were performed on an MTS machine equipped with a 100 N loading cell. The samples (D412-06a die A design scaled by 1/4, length × width × thickness = 14.75 × 3 × 0.3

mm) were elongated to a predetermined strain at deflection rate of 50 mm/min and recovered at a deflection rate of 5 mm/min at room temperature.

Pure shear test

A pure shear test was also used to characterize the toughness. Two different samples, notched and unnotched, were used to measure the tearing energy *T*. The samples were cut into a rectangular shape with a width of 10 mm and a length of 25 mm (a_0). The sample thickness was 0.42 mm (b_0). An initial notch of 12.5 mm in length was cut using a razor blade. The test piece was clamped on two sides, and the distance between the two clamps was fixed at 5 mm (L_0). The upper clamp was pulled upward at a constant speed of 100 mm/min, while the lower clamp was fixed. The force-length curves of the samples were recorded, and the tearing energy was calculated from $T = U(L_c)/(a_0 \times b_0)$, where $U(L_c)$ was the work done by the applied force to the unnotched sample at the critical stretching distance L_c. L_c was the distance between the two clamps when the crack starts to propagate in the notched sample. The onset of the crack propagation was determined using the movie image recorded by a camera.

***In vitro* degradation experiment**

The enzymatic degradation experiment was performed in the presence of lipase from *Thermomyces lanuginosus* (100,000 U/g, Sigma) at an activity of 2000 U/mL in 0.1 M Dulbecco's phosphate buffered saline (DPBS). A strip-shaped PSeD-U polymer sample (6 mm × 4 mm × 0.3 mm) was weighted, put in 5 mL of DPBS with lipase enzyme, and incubated at 37 °C. Every 2 h, the sample was retrieved, dried, and weighed. The degradation degree was determined by dry-weight change. For the PSeD-U20-12h bioelastomers samples, their mechanical properties were investigated by an Instron 5969 machine equipped with a 100 N loading cell during the degradation process. The samples were not tested to failure but a strain of 200%. So, the additional tensile test could be made on the same specimens in further degradation experiments. Three replicates were performed and averaged. The degradation experiment without lipase was performed in 0.1 M DPBS.

In vitro biocompatibility

Polymers solutions in 2,2,2-trifluoroethanol (1 g/L) were filtered through a 0.2 μm sterilization filter and added to 96-well tissue-culture polystyrene (TCPS) plates (20 μL/well). After evaporation of solvent in air, the plates were dried in a vacuum oven overnight and then washed with DPBS (Lonza, Walkersville, MD, USA) three times and culture medium once with gentle shaking, 30 min for each wash. Pristine TCPS were used as control groups. NIH 3T3 fibroblasts were seeded at a density of 5×10^3 cells/well on 96-well TCPS plates. The cells were cultured in Dulbecoo's modification Eagle's medium (DMEM; Mediatech, Manassas, VA, USA) with glutamine, containing 10% fetal bovine serum (Lonza) and 37 ^{o}C with 5% CO_2. After 2 days of incubation, cell viability (n = 5) was analyzed by a LIVE/DEAD Viability/Cytotoxicity Kit. The fluorescence images were taken using an inverted microscope Eclipse Ti (Nikon, Melville, NY, USA) equipped with a RETIGA-SRV digital camera (QImaging, Surrey, BC, Canada). Cell metabolic activity ($n \geq 4$) at seeding and 1 day, 3 days, and 5 days after seeding was determined by a Vybrant1 3-(4,5-dimethylthiazol-2-yl)-2,5-diphenyltetrazolium bromide (MTT) cell proliferation assay kit (Invitrogen). The absorbance was recorded by a SynergyMX plate reader (Biotek, Winooski, VT, USA).

In vivo biocompatibility

Subcutaneous implantation of samples in mice was used to evaluate the *in vivo* biocompatibility of PSeD-U20-12h elastomers. All animal experiments were conducted complying with guidelines of Institutional Animal Care and Use Committee (IACUC) at the Donghua University. BALB/c mice were commercially supplied by Slac Laboratory Animal Co. Ltd. (Shanghai, China) and divided into two groups (PLGA used as the control). The mice were initially anesthetized by using an intraperitoneal injection of 1% pentobarbital sodium (100 mg/kg) and the dorsum was shaved and cleaned with 2% iodine prior to the procedure. Then, sterilized samples were implanted into the subcutaneous pocket. At each predetermined time point (4, 7, and 28 days), the mice were killed and the implants with surrounding tissues were harvested. The specimens were

fixed with 4% paraformaldehyde for 48 h, dehydrated with a grade series of ethanol, embedded in paraffin, and cut into slices. The slices were stained with hematoxylin and eosin (H&E) for further analysis. Additionally, the weight changes of mice were recorded after subcutaneous implantation over the 28 days. The complete blood of the mice was also harvested to measure the white blood cells (WBC) concentration.

Statistical Analysis

Statistical analysis was performed using a one-way ANOVA test. A p value < 0.05 was considered significant. All values are reported as a mean ± standard deviation.

Piezocapacitive pressure sensor

The PSeD-U elastomers layer with 2D arrays of square pyramids that are formed from arrays of pyramidal recesses etched into the face of a Si-wafer mold. After being cured, the ~100 μm thick PSeD-U film was peeled off and laminated with the bottom and top electrodes. The electrodes were fabricated by casting a thin polycaprolactone (PCL) film, and evaporating gold (8-10 nm thick) after having exposed the substrate surface to oxygen plasma. The sensor was assembled by laminating the bottom electrode, intermediate PSeD-U dielectric layer, and top electrode. For applying vertical pressure to the sensor, a MTS machine equipped with a 25 N loading cell were utilized in the test. The capacitance changes from the applied pressure were obtained by a TH2832 LCR with software under AC voltages of 1 V at 200 kHz. To measure the capacitance changes under lateral pressure movements.

Strain sensor

The strain sensors were fabricated by spraying silver nanowires on the PSeD-U elastomers. 2 ml silver nanowires solution (CST-NW-S70, COLDSTONES TECH) were sprayed onto the PSeD-U20-12h elastomers films (3 x 3 cm^2) surface by a commercial airbrush with a distance of 20 cm. Then, the sprayed PSeD-U20-12h elastomers films dried at a temperature of 80 ^{o}C for 3 h. The square resistances of the conductive films at different strains were measured using Mitsubishi Chemical MCP-T370 handheld four points probe meter. A size of 3 x 0.5 cm^2 conductive films

were attached on the wrist back to monitoring the motion of human wrist. The resistance changes were recorded using a Keithley DMM7510 system electrometer.

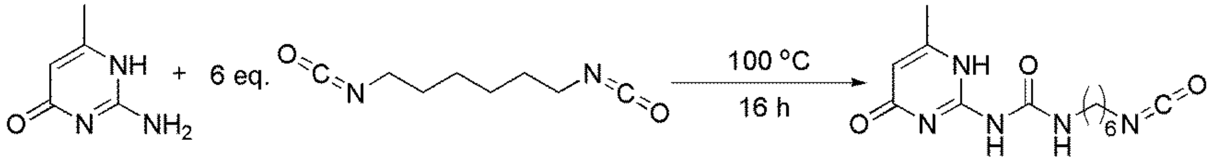

Supplementary Figure 1: Synthesis of UPy-HDI.

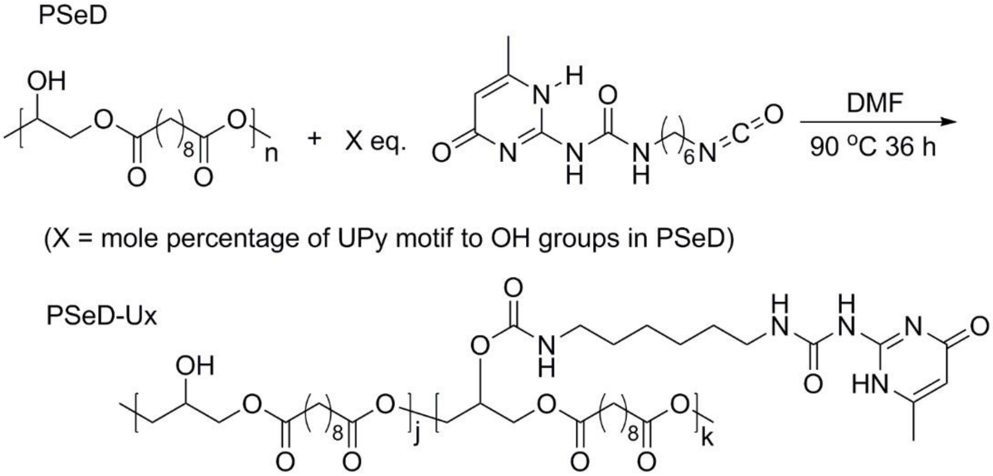

Supplementary Figure 2: Synthesis of PSeD-U polymers

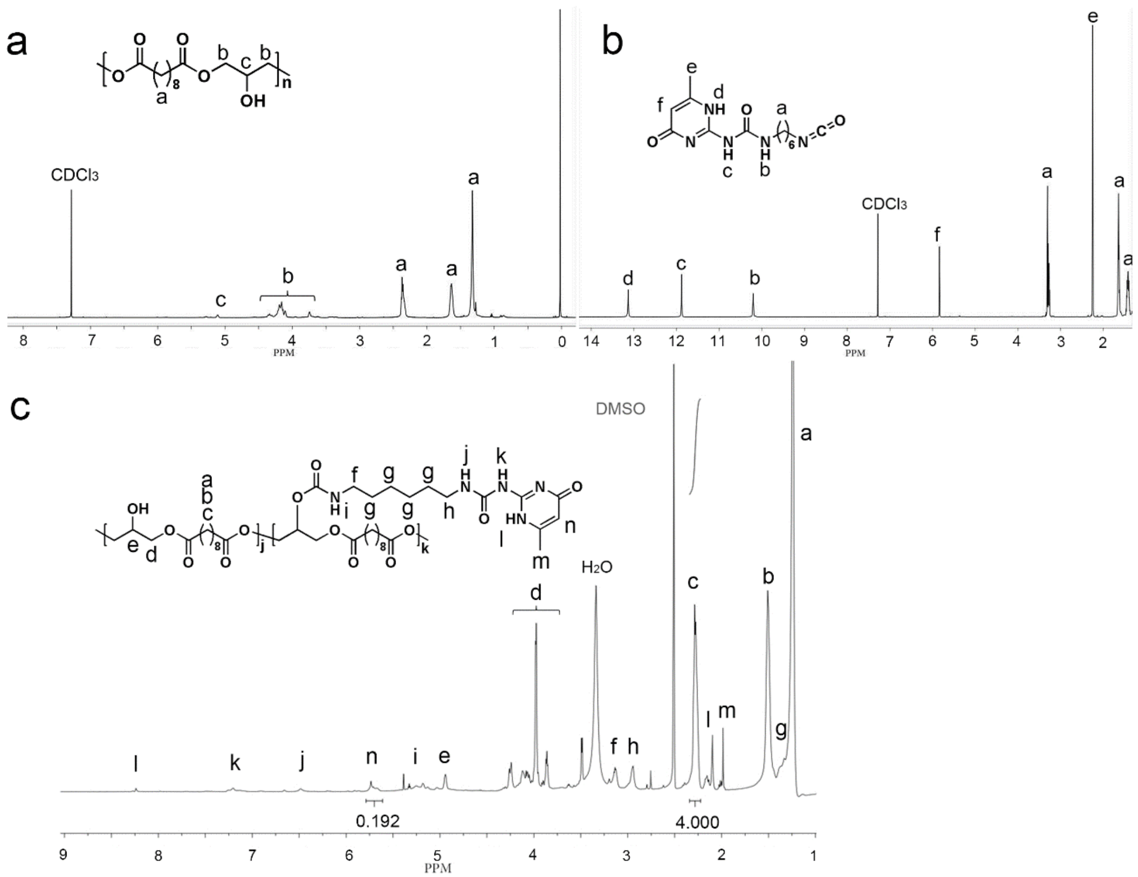

Supplementary Figure 3: ^{1}H NMR spectra of PSeD (a), UPy-HDI (b) and PSeD-U20 (c) polymer. The actual UPy contents in PSeD-U20 polymers were caluculated by comparing the relative proton integrations of the pyrimidyl groups in UPy at δ 5.71 ppm (the integration is 0.19) and the methylene groups in sebacate at δ 2.27 ppm (the intregration is 4.00).

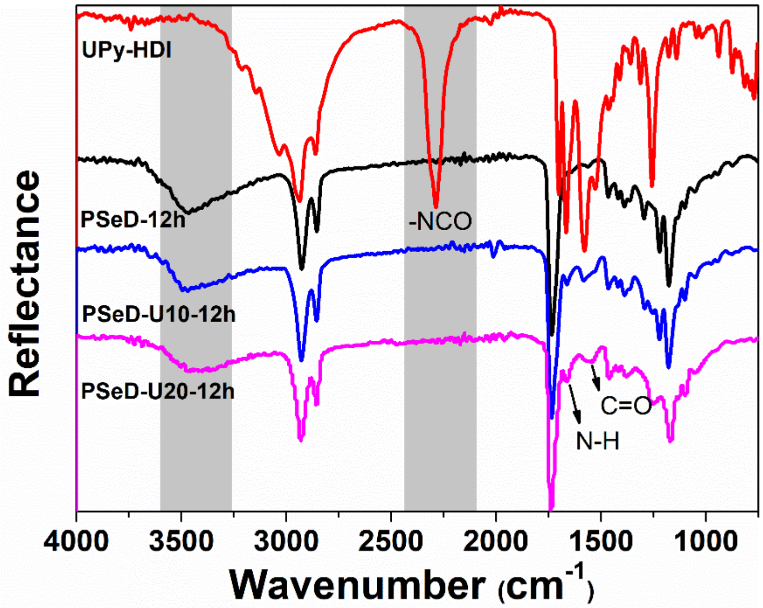

Supplementary Figure 4: FT-IR spectra of starting materials UPy-HDI and PSeD and PSeD-U elastomers. The absorption bands at 1583 and 1664 cm^{-1} corresponded to C=O bond and N-H bond in -CO-NH- groups, respectively, indicating the successful attachment of UPy groups to PSeD chains. The absence of isocyanate signal at 2285 cm^{-1} revealed the complete conversion of UPy-HDI. The broad peak of free hydroxyl groups at 3475 cm^{-1} became wider and shifted to lower wavenumbers with the increase of UPy groups, which revealed the presence of N-H bonds and strong hydrogen bonds in PSeD-U elastomers.

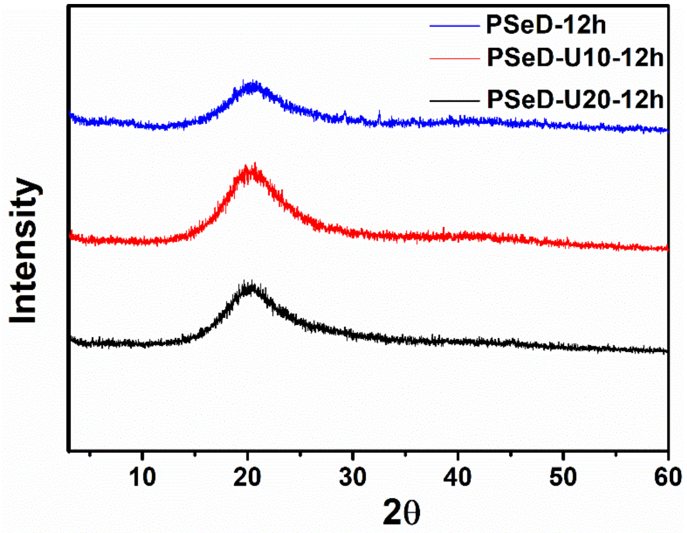

Supplementary Figure 5: The WAXS curves of PSeD and PSeD-U elastomers. The broad amorphous diffraction peaks at a 2θ of 21° suggested that all the three polymers are amorphous.

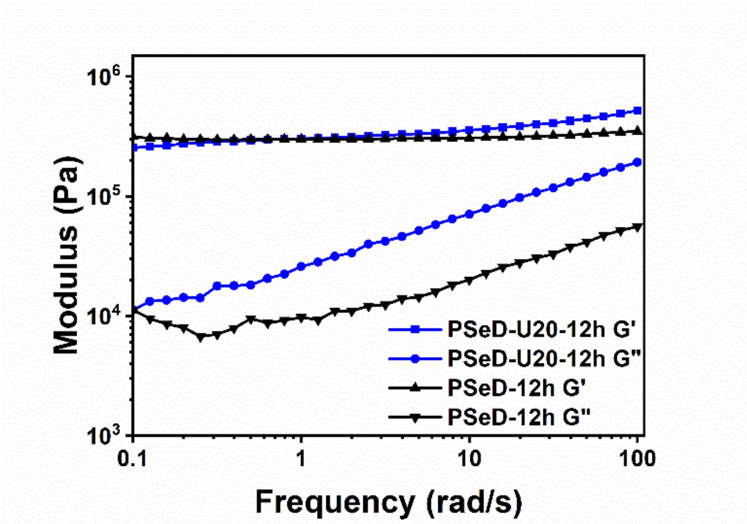

Supplementary Figure 6: The storage modulus G' and loss modulus G" of PSeD-12h and PSeD-U20-12h elastomers on frequency sweeps at 30 ^{o}C, demonstrating the hybrid crosslinked structure of PSeD-U20-12h elastomers.

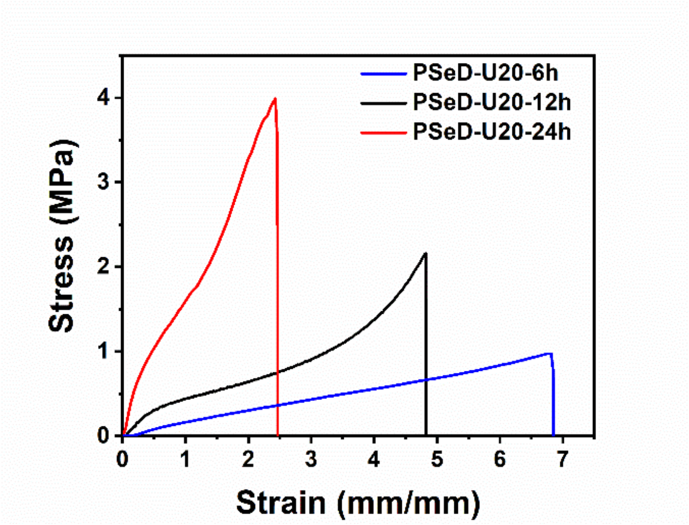

Supplementary Figure 7: Typical tensile stress-strain curves of PSeD-U20 elastomers with different densities of covalent crosslinks.

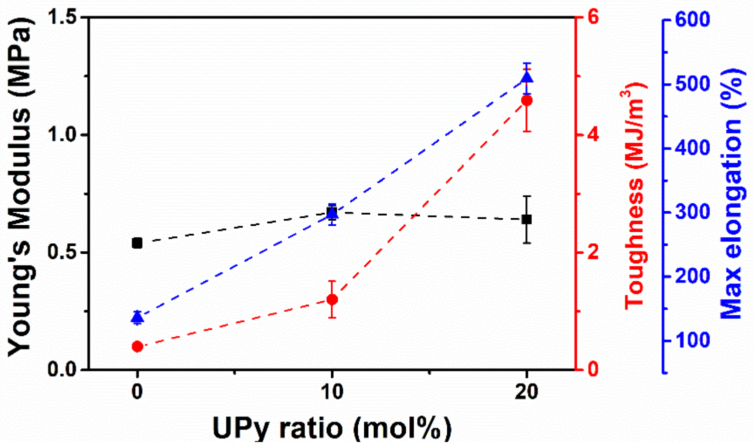

Supplementary Figure 8: The quantitative comparison of the mechanical properties of PSeD-U-12h elastomers (Figure 2a) with different densities of hydrogen bond while practically identical covalent crosslinking densities.

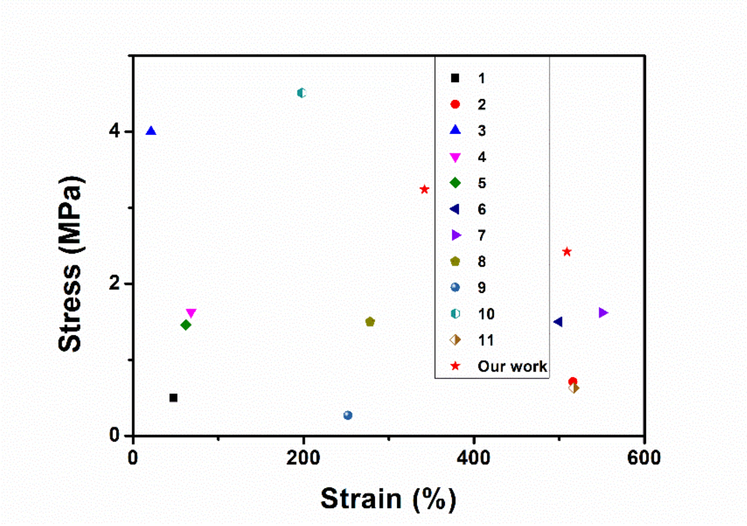

Supplementary Figure 9: The comparison of the strength of PSeD-U elastomers with previous reported PGS-derived elastomers. Reference: **1**, *Biomacromolecules*, 2007, 8, 3071; **2**, *Adv. Mater.*, 2013, 25, 1209; **3**, *J. Mater. Chem. B*, 2015, 3, 3222; **4**, *J. Appl. Polym. Sci.* 2015, 132, 42196; **5**, *Biomed. Mater*. 2009, 4, 025015; **6**, *Biomaterials* 2016, 104, 18; **7**, *Biomaterials* 2010, 31, 8516; **8**, *Acta Biomaterialia* 2018, 71, 279; **9**, *Polym. Chem.*, 2018, 9, 3727; **10**, *J. Mater. Chem. B*, 2019, 7, 3279; **11**, *ACS Omega* 2018, 3, 18714.

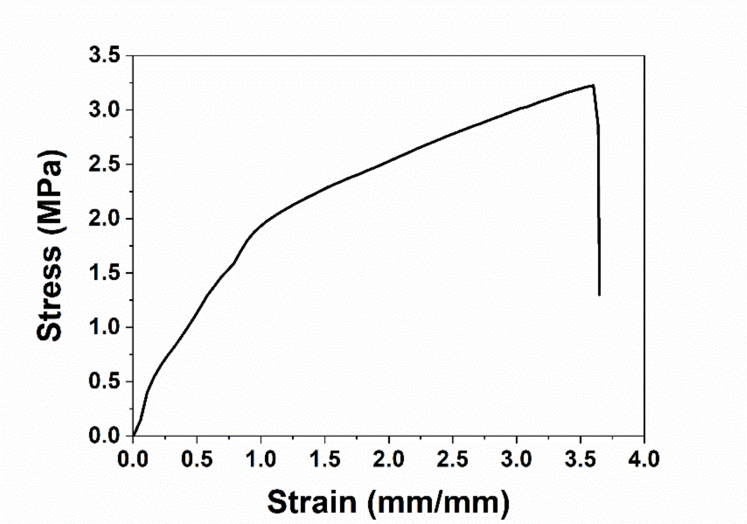

Supplementary Figure 10: Typical tensile stress-strain curves of PSeD-U30-12h elastomers.

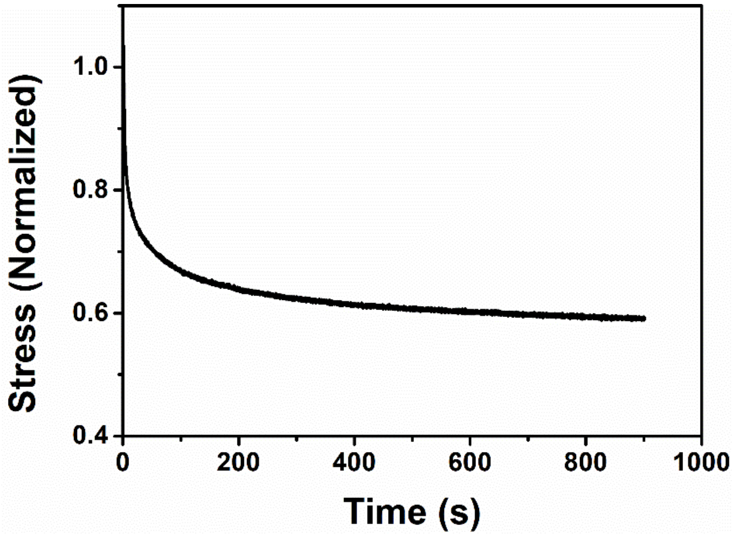

Supplementary Figure 11: The stress relaxation curves of PSeD-U20-12h elastomer stretched at 300% strain for 900 s. The applied force was apparently released due to the dissociation of the hydrogen bonds, exhibiting the energy dissipation of the hydrogen bonds.

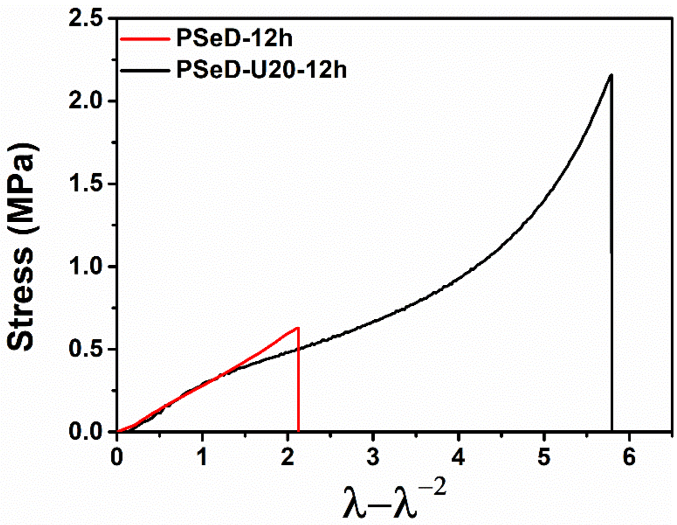

Supplementary Figure 12: The relationship between stress and extension ratio in uniaxial stretching expressed by classical Neo-Hookean model. The stress was plotted against the extension ratio ($\lambda - \lambda^{-2}$), the results are shown in Figure 2d. As for the uniaxial tension test, the extension ratio $\lambda = L/L_0$, where the L and L_0 are the length before and after stretching, respectively.

$$\sigma = \frac{\rho RT}{\overline{Mc}} (\lambda - \lambda^{-2})$$

Here, ρ, R, and T are the material density, the ideal gas constant, and the temperature, respectively. The is the average molecular weight between the crosslinks of the polymer chains. For the context of elasticity, the slope of the elastic deformation region equals shear modulus (G), which is $\rho RT/\overline{Mc}$ = G = 0.20 MPa. Since the elastomers with a Poisson ratio n = 0.5, the values of Young's modulus obtained from the relation E = 2G (1 + n) = 3G and from elastic deformation region of the strain-stress curve (0.60 MPa) are similar. The similar modulus also indicated the introduction of low concentration of hydrogen bonds would have little effect on the average molecular weight between the crosslinking points ($\overline{Mc}$).

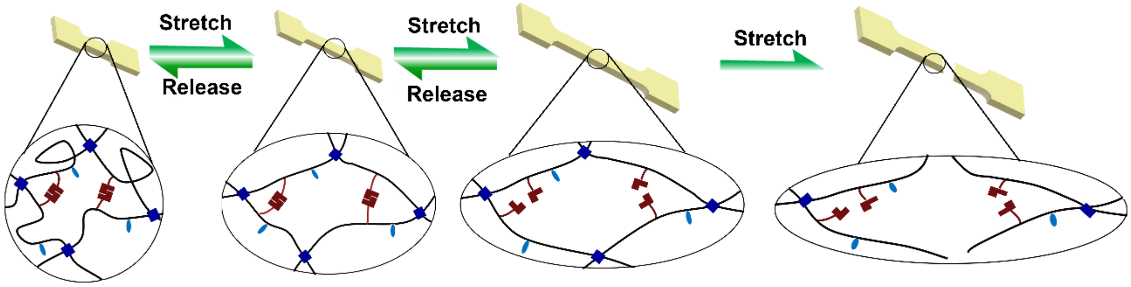

Supplementary Figure 13: Schematic of the evolution of molecular structures of PSeD-U elastomer during stretching, corresponding to the different regions of Figure 2c.

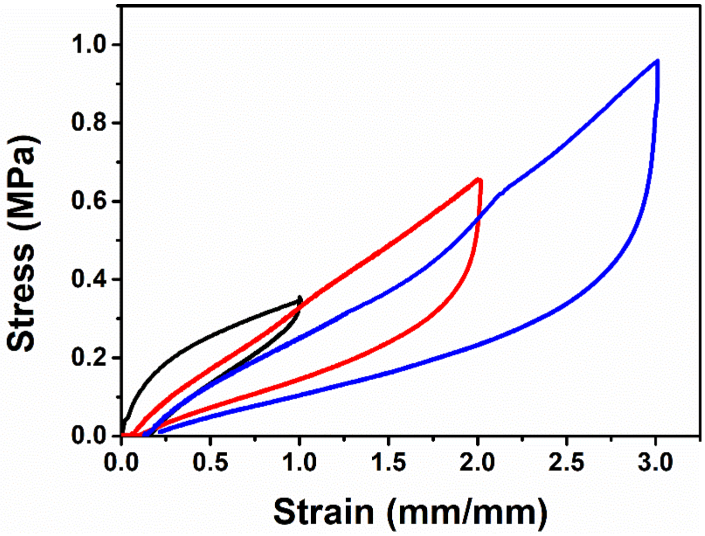

Supplementary Figure 14: The tensile stress-strain curves of loading-unloading cycles at different strains without waiting time between two consecutive loadings.

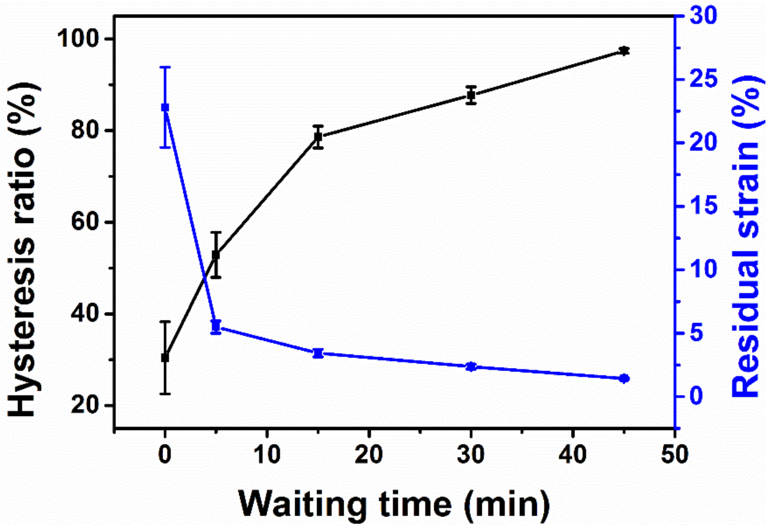

Supplementary Figure 15: The dependence of the hysteresis ratio (area ratio of the following cyclic loop to the first run) and residual strain on the waiting time of PSeD-U20-12h elastomers in Figure 3c.

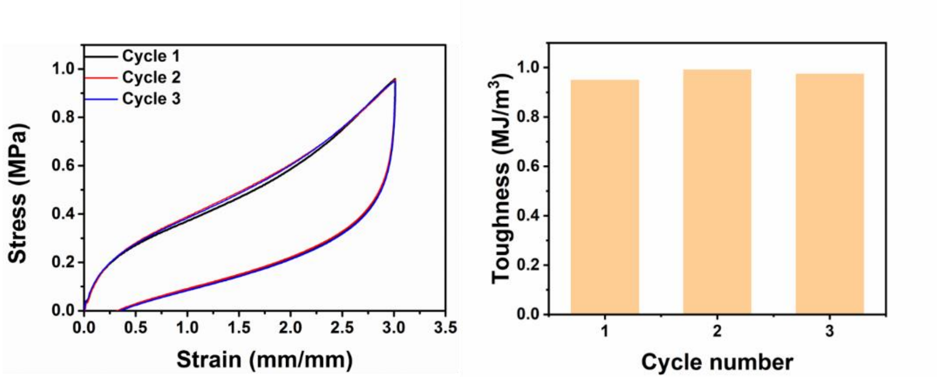

Supplementary Figure 16: Three cyclic tensile curves of PSeD-U20-12h elastomers with 1 h waiting time between each cycle (left) and the corresponding toughness (right). The high level of overlap between the original and subsequent curves indicated their robust recovery ability.

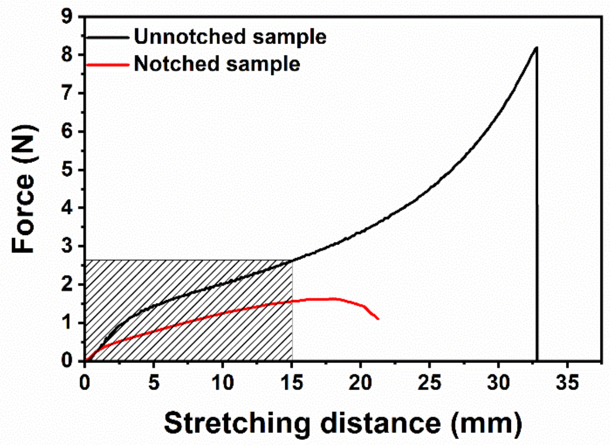

Supplementary Figure 17: The representative force-extension curves of the unnotched (black) and notched (red) samples of PSeD-U20-12h.

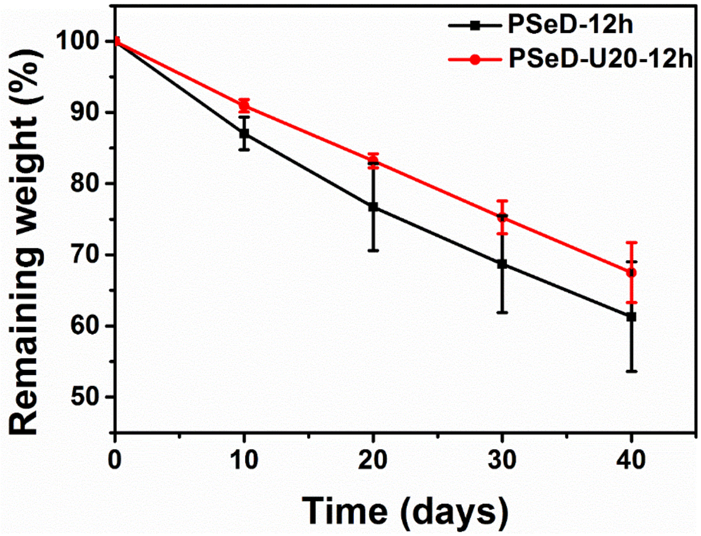

Supplementary Figure 18: *In vitro* degradation of PSeD and PSeD-U20-12h elastomers in DPBS solutions at 37 ^{o}C without lipase. PSeD-U20-12h elastomes degraded slower than PSeD elastomers likely due to the existing of hydrogen bonds in PSeD-U. Both elastomers showed a steady degradation with well-maintained rectangle shapes indicating a surface erosion mechanism favorable for *in vivo* applications.

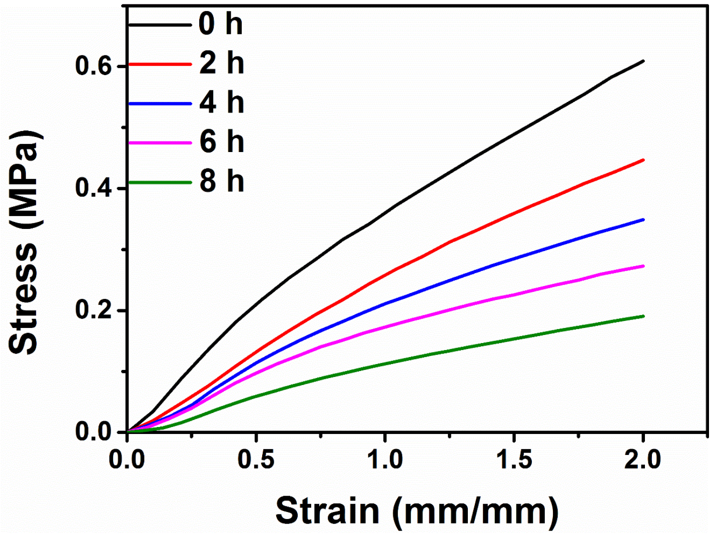

Supplementary Figure 19: Representative tensile stress-strain curves of PSeD-U20-12h elastomers after degradation in culture medium for 0, 2, 4, 6, 8 h. The tensile strength and Young's modulus decreased gradually with time, indicating the relatively mechanical stability of PSeD-U20-12h elastomers during the degradation.

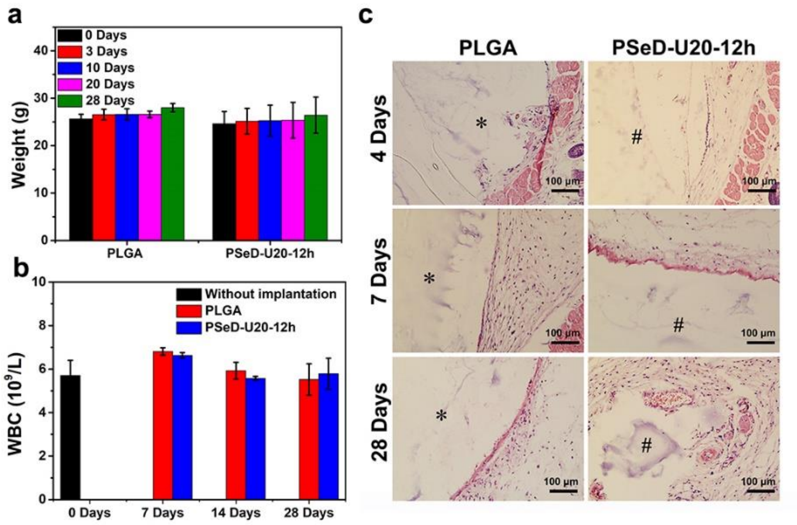

Supplementary Figure 20: *In vivo* biocompatibility of PSeD-U20-12h elastomers. a), The body weight of mice at different times after implantation. b), The WBC concentration of mice measured at different time points after implantation. c), Representative photomicrographs of H&E staining sections of retrieved PSeD-U20-12h (marked as #) and PLGA (marked as *) implants at different time points.

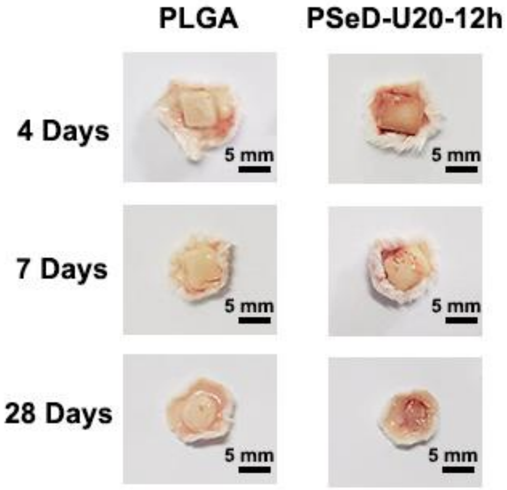

Supplementary Figure 21: *In vivo* biodegradability of PSeD-U20-12h elastomers. Representative photographs of retrieved PSeD-U20-12h and PLGA implants at different time points.

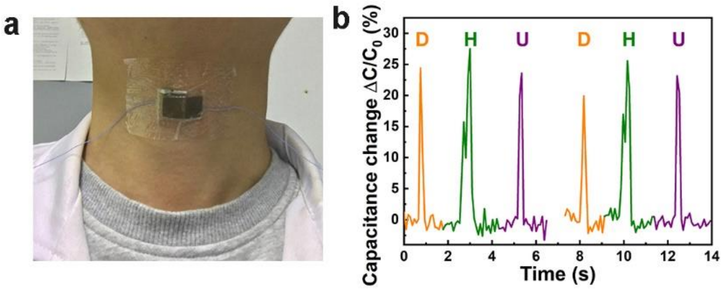

Supplementary Figure 22: The application of pressure sensors for phonation recognition. a), Representative photograph of pressure sensor attached on the throat. b), The capacitance change of the pressure sensor when person spoke phrase of “DHU”.

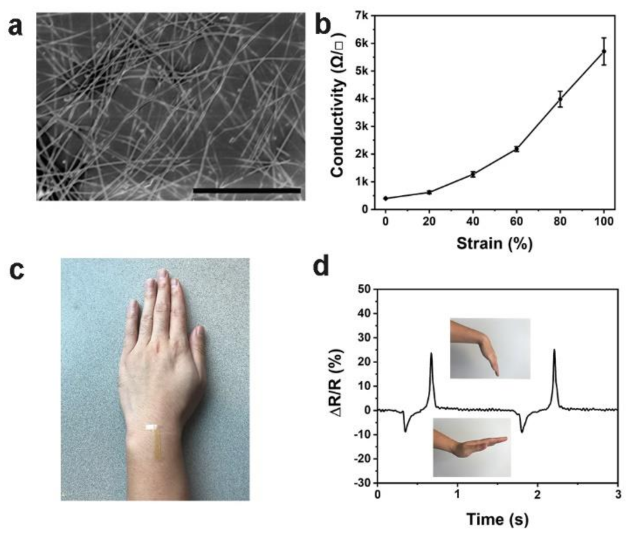

Supplementary Figure 23: The application of strain sensors based on PSeD-U elastomers. a), SEM of silver nanowires on PSeD-U20-12h elastomers. Scale car = 5 μm. b), Dependence of square resistance on strain for strain sensing. c), The photo of the strain sensor attached on the wrist. d), The change of resistance of the sensor attached on wrist during cyclic bending and stretching of wrist.

Supplementary Table 1: Molar contents of UPy groups in PSeD-U polymers and molecular weight of PSeD, PSeD-U polymers.

Materials	Molar contents of UPy groups[a]	M_n [kDa]	PDI
PSeD	-	10.0	1.33
PSeD-U10	9.8%	11.2	1.64
PSeD-U20	19.1%	12.0	1.81
PSeD-U30	28.9%	14.3	1.75

a, Experimental molar contents of UPy groups in PSeD-U polymers according to the relative proton integrations of the pyrimidyl groups in UPy at δ 5.71 ppm and the methylene groups in sebacate moiety at δ 2.27 ppm

Supplementary Table 2: The results of ICP tests and contents of UPy groups of PSeD-U polymers.

Materials	ICP results				Theoretical value	
	C (w%)	N (w%)	R (N/C)[a]	UPy%[b]	R (N/C)[a]	UPy%[b]
PSeD-U10	56.10%	1.93%	3.44%	9.83%	3.5%	10%
PSeD-U20	57.89%	3.57%	6.16%	19.25%	6.4%	20%
PSeD-U30	55.59%	4.77%	8.58%	29.25%	8.8%	30%

a, Molar ratio of N and C atoms
b, Molar content of UPy groups

Supplementary Table3: Mechanical properties of PSeD and PSeD-U elastomers

Materials	Young's modulus [MPa]	Strength [MPa]	Elongation [%]	Toughness [MJ/m^3]	Swelling ratio
PSeD-12h	0.57 ± 0.02	0.64 ± 0.03	136 ± 09	0.40 ± 0.02	3.45 ± 0.06
PSeD-U10-12h	0.67 ± 0.03	0.73 ± 0.10	297 ± 16	1.20 ± 0.31	3.39 ± 0.17
PSeD-U20-12h	0.64 ± 0.10	2.42 ± 0.54	509 ± 24	4.59 ± 0.53	3.37 ± 0.06
PSeD-U30-12h	1.49 ± 0.24	3.24 ± 0.45	342 ± 33	7.28 ± 0.95	3.30 ± 0.14
PSeD-U20-6h	0.23 ± 0.05	1.08 ± 0.16	662 ± 29	3.05 ± 0.62	5.43 ± 0.11
PSeD-U20-24h	2.85 ± 0.73	3.85 ± 0.43	258 ± 13	5.23 ± 0.73	2.37 ± 0.13
